# Role of the Ubiquitin Proteasome System in the Regulation of Blood Pressure: A Review

**DOI:** 10.3390/ijms21155358

**Published:** 2020-07-28

**Authors:** Osamu Yamazaki, Daigoro Hirohama, Kenichi Ishizawa, Shigeru Shibata

**Affiliations:** Division of Nephrology, Department of Internal Medicine, Teikyo University School of Medicine, Tokyo 173-8605, Japan; osamu195-tky@umin.ac.jp (O.Y.); hirohama-tky@umin.ac.jp (D.H.); ken.ishizawa@gmail.com (K.I.)

**Keywords:** blood pressure, renal salt reabsorption, vascular function, ubiquitin proteasome system

## Abstract

The kidney and the vasculature play crucial roles in regulating blood pressure. The ubiquitin proteasome system (UPS), a multienzyme process mediating covalent conjugation of the 76-amino acid polypeptide ubiquitin to a substrate protein followed by proteasomal degradation, is involved in multiple cellular processes by regulating protein turnover in various tissues. Increasing evidence demonstrates the roles of UPS in blood pressure regulation. In the kidney, filtered sodium is reabsorbed through diverse sodium transporters and channels along renal tubules, and studies conducted till date have provided insights into the complex molecular network through which ubiquitin ligases modulate sodium transport in different segments. Components of these pathways include ubiquitin ligase neuronal precursor cell-expressed developmentally downregulated 4-2, Cullin-3, and Kelch-like 3. Moreover, accumulating data indicate the roles of UPS in blood vessels, where it modulates nitric oxide bioavailability and vasoconstriction. Cullin-3 not only regulates renal salt reabsorption but also controls vascular tone using different adaptor proteins that target distinct substrates in vascular smooth muscle cells. In endothelial cells, UPS can also contribute to blood pressure regulation by modulating endothelial nitric oxide synthase. In this review, we summarize current knowledge regarding the role of UPS in blood pressure regulation, focusing on renal sodium reabsorption and vascular function.

## 1. Introduction

Hypertension is not only one of the most frequent diseases in the world, but it is also a key risk factor for cardiovascular disease and renal dysfunction. The kidney plays a pivotal role in the regulation of body fluid levels and blood pressure (BP), and an impaired kidney function comprises a major mechanism that alters the salt sensitivity of BP [1]. Because renal salt handling is critical for maintaining an independent life for terrestrial mammals, these animals have developed highly differentiated diverse tubule cells that are involved in the transport of sodium and other ions. The major renal sodium transporters and channels include Na^+^/H^+^ exchanger isoform 3 (NHE3) in the proximal tubule (PT), Na^+^-K^+^-2Cl^−^ cotransporter (NKCC2) in the thick ascending limb (TAL), Na^+^-Cl^−^ cotransporter (NCC) in the distal convoluted tubule (DCT), and epithelial sodium channel (ENaC) and Cl^−^/HCO_3_^−^ exchanger pendrin in the connecting tubule (CNT) and the collecting duct (CD). The significance of several of these transporters and their regulators in the renal nephron has been confirmed by the monogenic hypertensive or hypotensive disorders [2,3,4], as well as by the clinical efficacy of the pharmacological agents that block these sodium transport mechanisms.

In addition to the role of the kidney, it is well known that the dysregulation of vascular function significantly contributes to BP elevation [5,6]. The arterial wall consists of intimal endothelial cells, vascular smooth muscle cells, and adventitia. Vascular endothelial cells (VECs) play vital roles in regulating diverse biological functions by secreting various vasoactive factors, including nitric oxide (NO). NO, a strong vasodilator that tightly modulates vascular function, is primarily produced by endothelial NO synthase (eNOS) in endothelial cells [7]. Studies have demonstrated that both genetic and pharmacological ablation of eNOS elicits significant BP elevations [8,9,10]. Vascular smooth muscle cells (VSMCs) also play important roles in controlling the tonus of blood vessels, thereby regulating BP levels [11].

**Figure 1:**
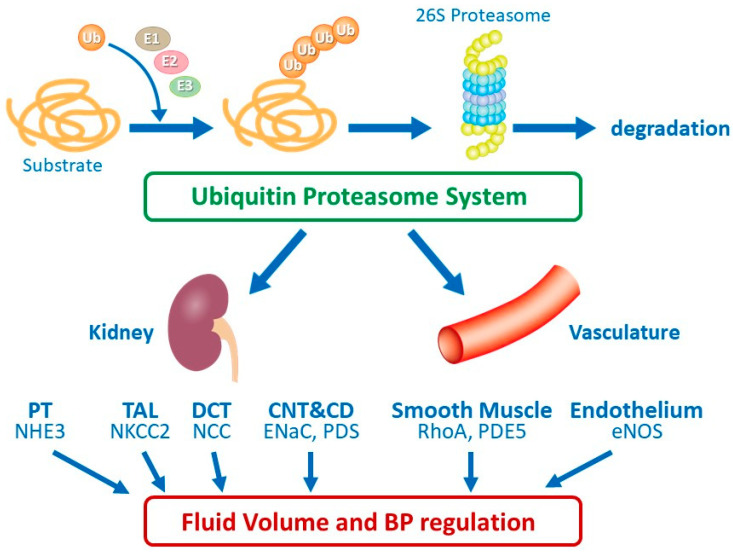
The role of ubiquitin proteasome system in fluid volume and blood pressure regulation. PT, proximal tubule; TAL, thick ascending limb; DCT, distal convoluted tubule; CNT, connecting tubule; CD, collecting duct.

Ubiquitylation is a stepwise process involving three classes of enzymes. Ubiquitin-activating enzymes (E1s) activate the ubiquitin molecule combined with ATP hydrolysis [12]. Ubiquitin is then transferred to ubiquitin-conjugating enzymes (E2s) with an active cysteine [13,14]. Following this, ubiquitin is transferred to substrates via the ubiquitin protein ligases (E3s). Humans have only one E1, ~40 E2s, and 500–1000 of E3s [15,16,17]. Two types of E3s exist, termed the homologous to the E6-AP C terminus and the really interesting new gene (RING). E3s provide substrate specificity to the ubiquitin system and recognize multiple substrates through different protein–protein interactions, thus regulating multiple cellular processes, including DNA damage repair, cell cycle progression, development, and signal transduction. Given that the ubiquitin proteasome system (UPS) enables adaptation to physiological challenges by controlling the protein abundance of target substrates, the involvement of UPS in BP regulation has attracted extensive research attention. In this article, we review the role of UPS in BP homeostasis, especially focusing on sodium transporters of the kidney and vascular functions (Figure 1).

## 2. Role of UPS in the Regulation of Tubular Function in the Kidney

### 2.1. Proximal Tubule

Among the salt transport mechanisms in the PT, NHE3 has a major role in sodium reabsorption in this segment [18,19]. Human NHE3 contains a PY motif that binds to ubiquitin ligase neuronal precursor cell-expressed developmentally downregulated 4-2 (NEDD4-2), and this interaction can modulate cell surface expression and internalization of NHE3 [20], although it is unclear whether NHE3 is directly ubiquitylated by Nedd4-2. It is interesting to note that this interaction appears to be exclusive to humans and several primates, which is because the PY motif in NHE3 was not identified in other mammals in the alignment analysis. Hatanaka et al. reported that angiotensin II signaling alters NHE3 levels, thereby regulating salt sensitivity [21]. Using subtotal nephrectomized mice, they showed that NHE3 abundance was lower in subtotal nephrectomized mice receiving azilsartan, an angiotensin II receptor 1 (AT1R) blocker, than in those receiving vehicle and that lactacystin, a proteasome inhibitor, blocked the azilsartan-induced decrease in NHE3 expression. These data indicate that NHE3 levels are regulated by UPS that are modulated by AT1R signaling. It currently remains unknown whether the interaction between NHE3 and NEDD4-2 is regulated by angiotensin II.

### 2.2. Thick Ascending Limb

NKCC2, a target of loop diuretics such as furosemide, regulates sodium reabsorption in the TAL [22]. Regarding the UPS-mediated modulation of NKCC2, Wu et al. reported a role of UPS in the regulation of NKCC2 abundance in a high-salt condition [23]. They used a cytochrome P450 4F2 transgenic mouse model, which exhibited an increased production of 20-hydroxyeicosatetraenoic acid (20-HETE), a regulator of vascular tone and renal sodium reabsorption, by blocking Ca^2+^-activated K^+^ channels [23]. Compared with wild-type mice, the transgenic mice displayed a profound decrease in renal NKCC2 abundance in response to a high-salt diet. This effect was not accompanied by the changes in NKCC2 mRNA expression but increased the abundance of ubiquitylated NKCC2. NKCC2 interacted with NEDD4-2, suggesting a role of this ubiquitin enzyme in the regulation of NKCC2 abundance. Another study reported that dibutyryl cyclic GMP (db-cGMP), a cell-permeable cGMP analog, decreased NKCC2 levels by increasing NKCC2 ubiquitylation and proteasomal degradation in rats [24]. In that study, db-cGMP induced a significant reduction in surface NKCC2 levels in suspensions of rat medullary TALs, which was inhibited in the presence of the proteasome inhibitor MG132. Furthermore, that study reported that NKCC2 levels were constitutively ubiquitylated and that the process was promoted by db-cGMP [24]. Pathways that modulate NKCC2 ubiquitylation at the downstream of cGMP signaling remains to be determined. Given that phosphorylation can regulate the interaction between substrates and ubiquitin ligases, roles of cGMP-dependent kinase might be worth exploring in future studies.

### 2.3. Distal Convoluted Tubule

#### 2.3.1. KLHL3-Mediated WNK4 Ubiquitylation and NCC

NCC, a target of thiazide diuretics, modulates sodium reabsorption in the DCT, and accumulating evidence has demonstrated its importance in controlling BP [25]. Familial hyperkalemic hypertension, also known as pseudohypoaldosteronism type II (PHAII) or Gordon syndrome, is characterized by salt-sensitive hypertension, hyperkalemia, and metabolic acidosis [26,27,28]. The phenotypes in these patients can be reversed by thiazide treatment, thus suggesting the involvement of NCC in the pathogenesis of PHAII. Till date, mutations in four genes are known to cause PHAII, which include serine-threonine kinase with-no-lysine (WNK) 1 and WNK4 and Cullin 3 (CUL3) and Kelch-like 3 (KLHL3), the components of the cullin-RING ubiquitin ligase (CRL) complex [3,28,29]. WNKs are substrates for the KLHL3-CUL3 ligase complex. WNKs phosphorylate the downstream kinases STE20/SPS1-related proline-alanine-rich protein kinase (SPAK) and oxidative stress-responsive 1 [30], which in turn increase the levels of phosphorylated NCC, an active form of NCC. We and others have identified by mass spectrometry and co-immunoprecipitation that KLHL3 normally binds to WNK1 and WNK4 [4,31,32,33]. KLHL3-WNK4 binding induces ubiquitylation in at least 15 specific sites, leading to reduced WNK4 levels [4]. KLHL3 is phosphorylated at serine 433 (KLHL3^S433^) in the Kelch domain, which is regulated by angiotensin II–protein kinase C signaling [34]. Of interest, this site is recurrently mutated in independent PHAII families, and phosphorylation or single amino acid substitution of this site impairs the binding of KLHL3 with WNKs, resulting in its accumulation and activation of downstream signaling. It has also been reported that Akt and protein kinase A (PKA), key downstream substrates of insulin and vasopressin signaling, respectively, increase phosphorylated KLHL3^S433^ [35]. In addition, calcium-sensing receptor signaling can modulate KLHL3-WNK4-SPAK pathway by phosphorylating KLHL3 and WNK4 [36,37]. Conversely, phosphatase calcineurin is capable of dephosphorylating KLHL3 phosphorylation at KLHL3^S433^ [38]. These mechanisms probably play important roles in several pathological conditions such as low-K^+^-induced BP elevation and hypertension associated with obese diabetes mellitus [39,40]. CRLs are activated by neddylation of cullin. It has been demonstrated that CUL3 is also neddylated, and that its neddylation status is regulated by multisubunit deneddylase COP9 signalosome [41,42].

#### 2.3.2. NEDD4-2-Mediated Ubiquitylation and NCC

Accumulating data also indicate that the ubiquitin ligase NEDD4-2 regulates NCC. Arroyo et al. demonstrated that in cultured cells, NEDD4-2 interacts with NCC, resulting in its ubiquitylation and reduced cell surface expression [43]. They also observed that serum/glucocorticoid-regulated kinase 1 (Sgk1) prevented the NEDD4-2-mediated deactivation of NCC in a kinase-dependent manner, indicating that Sgk1 is also involved in the NEDD4-2-mediated NCC regulation [43]. The role of Sgk1 in regulating NEDD4-2 and NCC has been demonstrated in vivo in Sgk1 knockout mice [44]. In another study, tetracycline-inducible, nephron-specific NEDD4-2 knockout mice exhibited increased NCC protein levels and salt-sensitive hypertension [45]. The mRNA expression of NCC remained unchanged, suggesting that NEDD4-2 regulates NCC abundance at the post-transcriptional level. Roy et al. reported that NEDD4-2 regulates NCC function through WNK1 [46]. They identified two alternatively spliced exons within a proline-rich region of WNK1 that contain PY motifs. NEDD4-2 binds to the PY motifs of WNK1, ubiquitylating WNK1 and targeting it for proteasomal degradation [46]. Dysregulation of NEDD4-2 has been implicated in the pathophysiology of salt-sensitive hypertension in a model of chronic kidney disease, which resulted in NCC activation through WNK1/SPAK [47]. In a recent study, Wu et al. reported that NEDD4-2 modulated NCC levels through a mechanism involving basolateral K^+^ channel Kir4.1 (KCNJ10) [48]. The authors observed that kidney-specific deletion of NEDD4-2 hyperpolarized the DCT membrane, accompanied by the increase in NCC abundance. These changes were abolished in kidney-specific NEDD4-2/KCNJ10 double-knockout mice, leading to the suppression of NCC and blunted thiazide-induced natriuresis [48]. These data demonstrate a role of Kir4.1 in the NEDD4-2-mediated regulation of NCC.

### 2.4. Connecting Tubule and Collecting Duct

#### 2.4.1. NEDD4-2-Mediated Ubiquitylation and ENaC

ENaC, consisting of three subunits, α, β, and γ, is a primary regulator of sodium reabsorption in the CNT and CD [49,50]. It has been reported that gain-of-function mutations of SCNN1B and SCNN1G cause Liddle’s syndrome, which is characterized by salt-sensitive hypertension, hypokalemia, metabolic alkalosis, and low aldosterone levels [51,52,53,54]. This phenotype is induced by the disruption or elimination of PY motifs in the β- and γ-subunits of ENaC. Provided that NEDD4-2 ubiquitylates ENaC and regulates its membrane expression and activity [55,56,57,58], these mutations cause both increased channel expression and intrinsic activity with a consequent increase of sodium reabsorption. When the renin-angiotensin-aldosterone system (RAAS) is inactivated, NEDD4-2 continuously ubiquitylates ENaC and downregulates ENaC abundance. When RAAS is activated, aldosterone-induced Sgk1 phosphorylates NEDD4-2, resulting in the recruitment of 14-3-3 adaptor proteins. These proteins inhibit the association between NEDD4-2 and ENaC, thereby leading to the elevation of ENaC levels [59,60]. Consistently, several studies have demonstrated that mice lacking functional NEDD4-2 exhibit high levels of ENaC and salt-sensitive hypertension [61,62]. In humans, several reports indicated that common variants in *NEDD4L* (encoding NEDD4-2) are associated with BP disorder [63,64,65,66].

#### 2.4.2. NEDD4-2 and Pendrin

Although there is limited information available regarding the role of UPS in the intercalated cells (ICs) of CNT and CD, a recent study has demonstrated a role of NEDD4-2 in regulating electrolyte transport mechanisms in these cells [67]. Nanami et al. examined the phenotype of IC-specific NEDD4-2 knockout mice and found that these mice displayed increased pendrin abundance and Cl^−^/HCO_3_^−^ transport in the ICs, accompanied by the elevation of BP [67]. Furthermore, pendrin gene ablation was found to eliminate the BP increase observed in global NEDD4-2 knockout mice. These data indicate that the ubiquitin ligase NEDD4-2 in ICs is also involved in electrolyte transport and regulation of BP.

## 3. Role of UPS in the Regulation of Vascular Function

### 3.1. Proteasome Inhibitors and Cardiovascular Disorders

It is well known that the vasculature is an important determinant of BP. UPS ubiquitously regulates tissue function and can regulate BP through its effect on blood vessels. Proteasome inhibitors have been clinically used as therapeutic agents for multiple myeloma. Carfilzomib, the first irreversible proteasome inhibitor, was found to bind selectively to its target, the chymotrypsin-like activity of the 20S proteasome [68]. It exhibited a higher efficacy in the treatment of patients with relapsed and/or refractory multiple myeloma when applied in combination with dexamethasone with or without lenalidomide [69,70]. Since its approval during the year 2010, there have been increasing reports of carfilzomib-associated cardiovascular adverse events, including hypertension. A systematic review and meta-analysis showed that hypertension (12.2%) was most common among carfilzomib-associated cardiovascular adverse events [71], supporting the involvement of UPS in BP control.

### 3.2. Vascular Endothelial Cells

With respect to the mechanisms of carfilzomib-associated hypertension, vascular endothelial dysfunction may play a vital role [71,72,73]. It is known that carfilzomib elicits renal toxic effects as well as microangiopathy, which is believed to be mediated by endothelial dysfunction [74,75,76]. The key feature of vascular endothelial dysfunction is the decreased NO bioavailability, which is caused due to low NO production and/or increased consumption. Provided that endothelial eNOS is responsible for most of the vascular NO produced [77], its dysfunction results in the impairment of endothelium-dependent vasodilatation [78]. Tetrahydrobiopterin (BH4) is known as an essential cofactor for eNOS-mediated NO synthesis [79]. GTP cyclohydrolase (GTPCH), the rate-limiting enzyme involved in BH4 synthesis, has been reported to be regulated by UPS, and that cigarette smoke extract diminished GTPCH abundance that was inhibited by the proteasomal inhibitor MG132 [80]. This BH4 depletion in turn induced eNOS uncoupling with the loss of NO generation and increased superoxide production, resulting in VEC dysfunction [80]. There are also data indicating that UPS-mediated degradation of GTPCH is associated with oxidative stress in angiotensin II-induced hypertension [81] and diabetes mellitus [82]. It was observed that angiotensin II induced the proteasomal degradation of GTPCH via tyrosine nitration of an important regulatory subunit of 26S proteasome, which was triggered by NADPH oxidase activation and generation of free radicals [81]. In another study, streptozotocin-induced diabetic mice displayed reduced eNOS activity, which was restored by the administration of a proteasome inhibitor through the inhibition of the proteasome-dependent GTPCH reduction [82]. These results imply that the UPS-mediated degradation of GTPCH underlies VCE dysfunction through eNOS regulation. In fact, there have been several reports demonstrating that proteasome inhibitors can improve the function of VECs [83,84,85]. The role of UPS in endothelial function may vary depending on the disease state and stage, and further studies are required to investigate the role of UPS in VECs.

### 3.3. Vascular Smooth Muscle Cells

The UPS in VSMCs can also regulate BP. Peroxisome proliferator-activated receptor gamma (PPARγ) is a nuclear regulator superfamily of transcription factors, which is an important regulator of lipid and glucose metabolism. PPARγ is expressed in numerous tissues, including VSMCs. Importantly, studies have shown that mutations (P467L or V290M) in the ligand-binding domain of PPARγ cause not only insulin resistance but also early-onset hypertension [86,87], indicating its role in BP regulation. Moreover, dominant negative mice model of PPARγ (S-P467L) in VSMCs developed arterial stiffness and vascular dysfunction, accompanied by hypertension [88,89]. These results indicate that PPARγ in VSMCs may play an essential role in regulating BP.

Recent studies have suggested that the effect of PPARγ in VSMCs is mediated by its downstream effector molecule, Rho-related BTB domain-containing protein 1 (RhoBTB1) [90]. RhoBTB1, a new subfamily of Rho GTPases [91], is expressed in various tissues [92]. Several genome-wide association studies have demonstrated that RhoBTB1 loci are associated with BP [93,94]. RhoBTB1 interacts with the N-terminal of CUL3 through its first BTB domain [95]. Recently, Mukohda et al. demonstrated that RhoBTB1 protects against hypertension and arterial stiffness by restoring the activity of phosphodiesterase 5 (PDE5) [89]. They generated tamoxifen-inducible and VSMC-specific RhoBTB1 transgenic mice (S-RhoBTB1) and found that Rho-BTB1 expression was reduced in S-P467L mice, whereas S-P467L/S-RhoBTB1 mice exhibited the restoration of RhoBTB1 expression and improvement of vasocontraction in VSMCs, which was accompanied by the reduced PDE5 activity, leading to the attenuation of hypertension. In addition, tadalafil, a PDE5 inhibitor, reduced BP in the S-P467L/S-RhoBTB1 mice. It is interesting to note that RhoBTB1 promoted PDE5 ubiquitylation in the presence of CUL3, which was blunted upon treatment with an inhibitor (MLN4924) of neddylation, a modification that is required for CUL3 activation. The authors concluded that RhoBTB1 is involved in the PPARγ-mediated regulation of BP by regulating PDE5 activity through CUL3-dependent ubiquitylation [89]. Accumulating data indicate that phosphodiesterase 3 (PDE3), another member of the phosphodiesterase family, also critically regulates BP. Recent studies have demonstrated that six missense mutations of PDE3A in six unrelated families with Mendelian hypertension exhibit severe salt-independent but age-dependent hypertension [96]. In vitro analyses of mesenchymal stem cell-derived VSMCs demonstrated that the mutations increased the PKA-mediated PDE3A phosphorylation and resulted in gain of function, with increased cAMP-hydrolytic activity [96]. Whether PDE3 is regulated through UPS needs to be determined.

CUL3 also mediates the ubiquitylation and degradation of RhoA by interacting with a BTB domain-containing adaptor, BACURD [97], which regulates vascular contraction. It has been demonstrated that hypertension-causing mutations in CUL3 impair RhoA ubiquitylation [98] and that selective expression of mutant CUL3 in VSMCs results in augmented RhoA signaling and vascular dysfunction, leading to elevation of BP [99,100].

## 4. Conclusions

In this review article, we have summarized the current evidence regarding the role of UPS in BP regulation, especially focusing on sodium reabsorption in the kidney and vascular functions (Figure 1). In the kidney, sodium reabsorption regulated by NEDD4-2 has been well characterized in principal cells and has been extensively analyzed in other nephron segments. Studies have also demonstrated the emerging roles of other mechanisms including CUL3 and KLHL3. In addition, accumulating evidence reveals the involvement of vascular functions in UPS-mediated BP regulation. Given that UPS is present ubiquitously and elicits multiple functions, future investigation is necessary for the complete elucidation of the precise role of UPS in modulating BP.

## Abbreviations

20-HETE	20-Hydroxyeicosatetraenoic acid
AT1R	Angiotensin II type 1 receptor
BH4	Tetrahydrobiopterin
BP	Blood pressure
CD	Collecting duct
CNT	Connecting tubule
CUL3	Cullin 3
DCT	Distal convoluted tubule
E1	Ubiquitin-activating enzyme
E2	Ubiquitin-conjugating enzyme
E3	Ubiquitin protein ligase
ENaC	Epithelial sodium channel
eNOS	Endothelial NO synthase
GTPCH	GTP cyclohydrolase
IC	Intercalated cell
KLHL3	Kelch-like 3
NCC	Na^+^-Cl^−^ cotransporter
NEDD4-2	Neuronal precursor cell-expressed developmentally downregulated 4-2
NHE3	Na^+^/H^+^ exchanger isoform 3
NKCC2	Na^+^-K^+^-2Cl^−^ cotransporter
NO	Nitric oxide
PDE	Phosphodiesterase
PHAII	Pseudohypoaldosteronism type II
PKA	Protein kinase A
PPARγ	Peroxisome proliferator-activated receptor gamma
PT	Proximal tubule
RhoBTB1	Rho-related BTB domain-containing protein 1
RING	Really interesting new gene
Sgk1	Serum/glucocorticoid-regulated kinase 1
SPAK	STE20/SPS1-related proline-alanine-rich protein kinase
TAL	Thick ascending limb
UPS	Ubiquitin proteasome system
VEC	Vascular endothelial cell
VSMC	Vascular smooth muscle cell
WNK	With-no-lysine

## References

[1] Guyton A.C. (1990). The surprising kidney-fluid mechanism for pressure control--its infinite gain!. Hypertension.

[2] Lifton R.P., Gharavi A.G., Geller D.S. (2001). Molecular mechanisms of human hypertension. Cell.

[3] Boyden L.M., Choi M., Choate K.A., Nelson-Williams C.J., Farhi A., Toka H.R., Tikhonova I.R., Bjornson R., Mane S.M., Colussi G. (2012). Mutations in kelch-like 3 and cullin 3 cause hypertension and electrolyte abnormalities. Nature.

[4] Shibata S., Zhang J., Puthumana J., Stone K.L., Lifton R.P. (2013). Kelch-like 3 and Cullin 3 regulate electrolyte homeostasis via ubiquitination and degradation of WNK4. Proc. Natl. Acad. Sci. USA.

[5] Linder L., Kiowski W., Bühler F.R., Lüscher T.F. (1990). Indirect evidence for release of endothelium-derived relaxing factor in human forearm circulation in vivo. Blunted response in essential hypertension. Circulation.

[6] Panza J.A., Quyyumi A.A., Brush J.E., Epstein S.E. (1990). Abnormal endothelium-dependent vascular relaxation in patients with essential hypertension. N. Engl. J. Med..

[7] Palmer R.M., Ashton D.S., Moncada S. (1988). Vascular endothelial cells synthesize nitric oxide from L-arginine. Nature.

[8] Huang P.L., Huang Z., Mashimo H., Bloch K.D., Moskowitz M.A., Bevan J.A., Fishman M.C. (1995). Hypertension in mice lacking the gene for endothelial nitric oxide synthase. Nature.

[9] Leonard A.M., Chafe L.L., Montani J.P., Van Vliet B.N. (2006). Increased salt-sensitivity in endothelial nitric oxide synthase-knockout mice. Am. J. Hypertens.

[10] Lahera V., Salom M.G., Miranda-Guardiola F., Moncada S., Romero J.C. (1991). Effects of NG-nitro-L-arginine methyl ester on renal function and blood pressure. Am. J. Physiol..

[11] Owens G.K., Kumar M.S., Wamhoff B.R. (2004). Molecular regulation of vascular smooth muscle cell differentiation in development and disease. Physiol. Rev..

[12] Haas A.L., Warms J.V., Rose I.A. (1983). Ubiquitin adenylate: Structure and role in ubiquitin activation. Biochemistry.

[13] Jentsch S. (1992). The ubiquitin-conjugation system. Annu. Rev. Genet..

[14] Hershko A., Ciechanover A. (1998). The ubiquitin system. Annu. Rev. Biochem..

[15] Clague M.J., Heride C., Urbé S. (2015). The demographics of the ubiquitin system. Trends Cell Biol..

[16] Van Wijk S.J., Timmers H.T. (2010). The family of ubiquitin-conjugating enzymes (E2s): Deciding between life and death of proteins. FASEB J..

[17] Nakayama K.I., Nakayama K. (2006). Ubiquitin ligases: Cell-cycle control and cancer. Nat. Rev. Cancer.

[18] Kocinsky H.S., Girardi A.C., Biemesderfer D., Nguyen T., Mentone S., Orlowski J., Aronson P.S. (2005). Use of phospho-specific antibodies to determine the phosphorylation of endogenous Na+/H+ exchanger NHE3 at PKA consensus sites. Am. J. Physiol. Renal Physiol..

[19] Thomson R.B., Wang T., Thomson B.R., Tarrats L., Girardi A., Mentone S., Soleimani M., Kocher O., Aronson P.S. (2005). Role of PDZK1 in membrane expression of renal brush border ion exchangers. Proc. Natl. Acad. Sci. USA.

[20] No Y.R., He P., Yoo B.K., Yun C.C. (2014). Unique regulation of human Na+/H+ exchanger 3 (NHE3) by Nedd4-2 ligase that differs from non-primate NHE3s. J. Biol. Chem..

[21] Hatanaka M., Kaimori J.Y., Yamamoto S., Matsui I., Hamano T., Takabatake Y., Ecelbarger C.M., Takahara S., Isaka Y., Rakugi H. (2016). Azilsartan Improves Salt Sensitivity by Modulating the Proximal Tubular Na+-H+ Exchanger-3 in Mice. PLoS ONE.

[22] Russell J.M. (2000). Sodium-potassium-chloride cotransport. Physiol. Rev..

[23] Wu J., Liu X., Lai G., Yang X., Wang L., Zhao Y. (2013). Synergistical effect of 20-HETE and high salt on NKCC2 protein and blood pressure via ubiquitin-proteasome pathway. Hum. Genet..

[24] Ares G.R. (2019). cGMP induces degradation of NKCC2 in the thick ascending limb via the ubiquitin-proteasomal system. Am. J. Physiol. Renal Physiol..

[25] Gamba G. (2009). The thiazide-sensitive Na+-Cl- cotransporter: Molecular biology, functional properties, and regulation by WNKs. Am. J. Physiol. Renal Physiol..

[26] Mayan H., Munter G., Shaharabany M., Mouallem M., Pauzner R., Holtzman E.J., Farfel Z. (2004). Hypercalciuria in familial hyperkalemia and hypertension accompanies hyperkalemia and precedes hypertension: Description of a large family with the Q565E WNK4 mutation. J. Clin. Endocrinol. Metab..

[27] Mayan H., Vered I., Mouallem M., Tzadok-Witkon M., Pauzner R., Farfel Z. (2002). Pseudohypoaldosteronism type II: Marked sensitivity to thiazides, hypercalciuria, normomagnesemia, and low bone mineral density. J. Clin. Endocrinol. Metab..

[28] Wilson F.H., Disse-Nicodeme S., Choate K.A., Ishikawa K., Nelson-Williams C., Desitter I., Gunel M., Milford D.V., Lipkin G.W., Achard J.M. (2001). Human hypertension caused by mutations in WNK kinases. Science.

[29] Louis-Dit-Picard H., Barc J., Trujillano D., Miserey-Lenkei S., Bouatia-Naji N., Pylypenko O., Beaurain G., Bonnefond A., Sand O., Simian C. (2012). KLHL3 mutations cause familial hyperkalemic hypertension by impairing ion transport in the distal nephron. Nat. Genet..

[30] Vitari A.C., Deak M., Morrice N.A., Alessi D.R. (2005). The WNK1 and WNK4 protein kinases that are mutated in Gordon’s hypertension syndrome phosphorylate and activate SPAK and OSR1 protein kinases. Biochem. J..

[31] Ohta A., Schumacher F.R., Mehellou Y., Johnson C., Knebel A., Macartney T.J., Wood N.T., Alessi D.R., Kurz T. (2013). The CUL3-KLHL3 E3 ligase complex mutated in Gordon’s hypertension syndrome interacts with and ubiquitylates WNK isoforms: Disease-causing mutations in KLHL3 and WNK4 disrupt interaction. Biochem. J..

[32] Wakabayashi M., Mori T., Isobe K., Sohara E., Susa K., Araki Y., Chiga M., Kikuchi E., Nomura N., Mori Y. (2013). Impaired KLHL3-mediated ubiquitination of WNK4 causes human hypertension. Cell Rep..

[33] Wu G., Peng J.B. (2013). Disease-causing mutations in KLHL3 impair its effect on WNK4 degradation. FEBS Lett..

[34] Shibata S., Arroyo J.P., Castaneda-Bueno M., Puthumana J., Zhang J., Uchida S., Stone K.L., Lam T.T., Lifton R.P. (2014). Angiotensin II signaling via protein kinase C phosphorylates Kelch-like 3, preventing WNK4 degradation. Proc. Natl. Acad. Sci. USA.

[35] Yoshizaki Y., Mori Y., Tsuzaki Y., Mori T., Nomura N., Wakabayashi M., Takahashi D., Zeniya M., Kikuchi E., Araki Y. (2015). Impaired degradation of WNK by Akt and PKA phosphorylation of KLHL3. Biochem. Biophys. Res. Commun..

[36] Castañeda-Bueno M., Arroyo J.P., Zhang J., Puthumana J., Yarborough O., Shibata S., Rojas-Vega L., Gamba G., Rinehart J., Lifton R.P. (2017). Phosphorylation by PKC and PKA regulate the kinase activity and downstream signaling of WNK4. Proc. Natl. Acad. Sci. USA.

[37] Bazúa-Valenti S., Rojas-Vega L., Castañeda-Bueno M., Barrera-Chimal J., Bautista R., Cervantes-Pérez L.G., Vázquez N., Plata C., Murillo-de-Ozores A.R., González-Mariscal L. (2018). The Calcium-Sensing Receptor Increases Activity of the Renal NCC through the WNK4-SPAK Pathway. J. Am. Soc. Nephrol..

[38] Ishizawa K., Wang Q., Li J., Yamazaki O., Tamura Y., Fujigaki Y., Uchida S., Lifton R.P., Shibata S. (2019). Calcineurin dephosphorylates Kelch-like 3, reversing phosphorylation by angiotensin II and regulating renal electrolyte handling. Proc. Natl. Acad. Sci. USA.

[39] Ishizawa K., Xu N., Loffing J., Lifton R.P., Fujita T., Uchida S., Shibata S. (2016). Potassium depletion stimulates Na-Cl cotransporter via phosphorylation and inactivation of the ubiquitin ligase Kelch-like 3. Biochem. Biophys. Res. Commun..

[40] Ishizawa K., Wang Q., Li J., Xu N., Nemoto Y., Morimoto C., Fujii W., Tamura Y., Fujigaki Y., Tsukamoto K. (2019). Inhibition of Sodium Glucose Cotransporter 2 Attenuates the Dysregulation of Kelch-Like 3 and NaCl Cotransporter in Obese Diabetic Mice. J. Am. Soc. Nephrol..

[41] McCormick J.A., Yang C.L., Zhang C., Davidge B., Blankenstein K.I., Terker A.S., Yarbrough B., Meermeier N.P., Park H.J., McCully B. (2014). Hyperkalemic hypertension-associated cullin 3 promotes WNK signaling by degrading KLHL3. J. Clin. Invest..

[42] Cornelius R.J., Si J., Cuevas C.A., Nelson J.W., Gratreak B.D.K., Pardi R., Yang C.L., Ellison D.H. (2018). Renal COP9 Signalosome Deficiency Alters CUL3-KLHL3-WNK Signaling Pathway. J. Am. Soc. Nephrol..

[43] Arroyo J.P., Lagnaz D., Ronzaud C., Vazquez N., Ko B.S., Moddes L., Ruffieux-Daidie D., Hausel P., Koesters R., Yang B. (2011). Nedd4-2 modulates renal Na+-Cl- cotransporter via the aldosterone-SGK1-Nedd4-2 pathway. J. Am. Soc. Nephrol..

[44] Faresse N., Lagnaz D., Debonneville A., Ismailji A., Maillard M., Fejes-Toth G., Náray-Fejes-Tóth A., Staub O. (2012). Inducible kidney-specific Sgk1 knockout mice show a salt-losing phenotype. Am. J. Physiol. Renal Physiol..

[45] Ronzaud C., Loffing-Cueni D., Hausel P., Debonneville A., Malsure S.R., Fowler-Jaeger N., Boase N.A., Perrier R., Maillard M., Yang B. (2013). Renal tubular NEDD4-2 deficiency causes NCC-mediated salt-dependent hypertension. J. Clin. Invest..

[46] Roy A., Al-Qusairi L., Donnelly B.F., Ronzaud C., Marciszyn A.L., Gong F., Chang Y.P., Butterworth M.B., Pastor-Soler N.M., Hallows K.R. (2015). Alternatively spliced proline-rich cassettes link WNK1 to aldosterone action. J. Clin. Invest..

[47] Furusho T., Sohara E., Mandai S., Kikuchi H., Takahashi N., Fujimaru T., Hashimoto H., Arai Y., Ando F., Zeniya M. (2020). Renal TNFα activates the WNK phosphorylation cascade and contributes to salt-sensitive hypertension in chronic kidney disease. Kidney Int..

[48] Wu P., Su X.T., Gao Z.X., Zhang D.D., Duan X.P., Xiao Y., Staub O., Wang W.H., Lin D.H. (2020). Renal Tubule Nedd4-2 Deficiency Stimulates Kir4.1/Kir5.1 and Thiazide-Sensitive NaCl Cotransporter in Distal Convoluted Tubule. J. Am. Soc. Nephrol..

[49] Rossier B.C. (2014). Epithelial sodium channel (ENaC) and the control of blood pressure. Curr. Opin. Pharmacol..

[50] Pearce D., Soundararajan R., Trimpert C., Kashlan O.B., Deen P.M., Kohan D.E. (2015). Collecting duct principal cell transport processes and their regulation. Clin. J. Am. Soc. Nephrol..

[51] Schild L., Canessa C.M., Shimkets R.A., Gautschi I., Lifton R.P., Rossier B.C. (1995). A mutation in the epithelial sodium channel causing Liddle disease increases channel activity in the Xenopus laevis oocyte expression system. Proc. Natl. Acad. Sci. USA.

[52] Hansson J.H., Nelson-Williams C., Suzuki H., Schild L., Shimkets R., Lu Y., Canessa C., Iwasaki T., Rossier B., Lifton R.P. (1995). Hypertension caused by a truncated epithelial sodium channel gamma subunit: Genetic heterogeneity of Liddle syndrome. Nat. Genet..

[53] Tamura H., Schild L., Enomoto N., Matsui N., Marumo F., Rossier B.C. (1996). Liddle disease caused by a missense mutation of beta subunit of the epithelial sodium channel gene. J. Clin. Invest..

[54] Staub O., Dho S., Henry P., Correa J., Ishikawa T., McGlade J., Rotin D. (1996). WW domains of Nedd4 bind to the proline-rich PY motifs in the epithelial Na+ channel deleted in Liddle’s syndrome. EMBO J..

[55] Staub O., Gautschi I., Ishikawa T., Breitschopf K., Ciechanover A., Schild L., Rotin D. (1997). Regulation of stability and function of the epithelial Na+ channel (ENaC) by ubiquitination. EMBO J..

[56] Lu C., Pribanic S., Debonneville A., Jiang C., Rotin D. (2007). The PY motif of ENaC, mutated in Liddle syndrome, regulates channel internalization, sorting and mobilization from subapical pool. Traffic.

[57] Wiemuth D., Ke Y., Rohlfs M., McDonald F.J. (2007). Epithelial sodium channel (ENaC) is multi-ubiquitinated at the cell surface. Biochem. J..

[58] Zhou R., Patel S.V., Snyder P.M. (2007). Nedd4-2 catalyzes ubiquitination and degradation of cell surface ENaC. J. Biol. Chem..

[59] Lang F., Pearce D. (2016). Regulation of the epithelial Na+ channel by the mTORC2/SGK1 pathway. Nephrol. Dial. Transplant..

[60] Bhalla V., Daidie D., Li H., Pao A.C., LaGrange L.P., Wang J., Vandewalle A., Stockand J.D., Staub O., Pearce D. (2005). Serum- and glucocorticoid-regulated kinase 1 regulates ubiquitin ligase neural precursor cell-expressed, developmentally down-regulated protein 4-2 by inducing interaction with 14-3-3. Mol. Endocrinol..

[61] Shi P.P., Cao X.R., Sweezer E.M., Kinney T.S., Williams N.R., Husted R.F., Nair R., Weiss R.M., Williamson R.A., Sigmund C.D. (2008). Salt-sensitive hypertension and cardiac hypertrophy in mice deficient in the ubiquitin ligase Nedd4-2. Am. J. Physiol. Renal Physiol..

[62] Minegishi S., Ishigami T., Kino T., Chen L., Nakashima-Sasaki R., Araki N., Yatsu K., Fujita M., Umemura S. (2016). An isoform of Nedd4-2 is critically involved in the renal adaptation to high salt intake in mice. Sci. Rep..

[63] Dunn D.M., Ishigami T., Pankow J., von Niederhausern A., Alder J., Hunt S.C., Leppert M.F., Lalouel J.M., Weiss R.B. (2002). Common variant of human NEDD4L activates a cryptic splice site to form a frameshifted transcript. J. Hum. Genet..

[64] Fouladkou F., Alikhani-Koopaei R., Vogt B., Flores S.Y., Malbert-Colas L., Lecomte M.C., Loffing J., Frey F.J., Frey B.M., Staub O. (2004). A naturally occurring human Nedd4-2 variant displays impaired ENaC regulation in Xenopus laevis oocytes. Am. J. Physiol. Renal Physiol..

[65] Araki N., Umemura M., Miyagi Y., Yabana M., Miki Y., Tamura K., Uchino K., Aoki R., Goshima Y., Umemura S. (2008). Expression, transcription, and possible antagonistic interaction of the human Nedd4L gene variant: Implications for essential hypertension. Hypertension.

[66] Luo F., Wang Y., Wang X., Sun K., Zhou X., Hui R. (2009). A functional variant of NEDD4L is associated with hypertension, antihypertensive response, and orthostatic hypotension. Hypertension.

[67] Nanami M., Pham T.D., Kim Y.H., Yang B., Sutliff R.L., Staub O., Klein J.D., Lopez-Cayuqueo K.I., Chambrey R., Park A.Y. (2018). The Role of Intercalated Cell Nedd4-2 in BP Regulation, Ion Transport, and Transporter Expression. J. Am. Soc. Nephrol..

[68] Kuhn D.J., Chen Q., Voorhees P.M., Strader J.S., Shenk K.D., Sun C.M., Demo S.D., Bennett M.K., van Leeuwen F.W., Chanan-Khan A.A. (2007). Potent activity of carfilzomib, a novel, irreversible inhibitor of the ubiquitin-proteasome pathway, against preclinical models of multiple myeloma. Blood.

[69] Stewart A.K., Rajkumar S.V., Dimopoulos M.A., Masszi T., Špička I., Oriol A., Hájek R., Rosiñol L., Siegel D.S., Mihaylov G.G. (2015). Carfilzomib, lenalidomide, and dexamethasone for relapsed multiple myeloma. N. Engl. J. Med..

[70] Dimopoulos M.A., Moreau P., Palumbo A., Joshua D., Pour L., Hájek R., Facon T., Ludwig H., Oriol A., Goldschmidt H. (2016). Carfilzomib and dexamethasone versus bortezomib and dexamethasone for patients with relapsed or refractory multiple myeloma (ENDEAVOR): A randomised, phase 3, open-label, multicentre study. Lancet Oncol..

[71] Waxman A.J., Clasen S., Hwang W.T., Garfall A., Vogl D.T., Carver J., O’Quinn R., Cohen A.D., Stadtmauer E.A., Ky B. (2018). Carfilzomib-Associated Cardiovascular Adverse Events: A Systematic Review and Meta-analysis. JAMA Oncol..

[72] Gavazzoni M., Vizzardi E., Gorga E., Bonadei I., Rossi L., Belotti A., Rossi G., Ribolla R., Metra M., Raddino R. (2018). Mechanism of cardiovascular toxicity by proteasome inhibitors: New paradigm derived from clinical and pre-clinical evidence. Eur. J. Pharmacol..

[73] Chen-Scarabelli C., Corsetti G., Pasini E., Dioguardi F.S., Sahni G., Narula J., Gavazzoni M., Patel H., Saravolatz L., Knight R. (2017). Spasmogenic Effects of the Proteasome Inhibitor Carfilzomib on Coronary Resistance, Vascular Tone and Reactivity. EBioMedicine.

[74] Rosenthal A., Luthi J., Belohlavek M., Kortüm K.M., Mookadam F., Mayo A., Fonseca R., Bergsagel P.L., Reeder C.B., Mikhael J.R. (2016). Carfilzomib and the cardiorenal system in myeloma: An endothelial effect?. Blood Cancer J..

[75] Yui J.C., Van Keer J., Weiss B.M., Waxman A.J., Palmer M.B., D’Agati V.D., Kastritis E., Dimopoulos M.A., Vij R., Bansal D. (2016). Proteasome inhibitor associated thrombotic microangiopathy. Am. J. Hematol..

[76] Hobeika L., Self S.E., Velez J.C. (2014). Renal thrombotic microangiopathy and podocytopathy associated with the use of carfilzomib in a patient with multiple myeloma. BMC Nephrol..

[77] Förstermann U., Münzel T. (2006). Endothelial nitric oxide synthase in vascular disease: From marvel to menace. Circulation.

[78] Vanhoutte P.M., Zhao Y., Xu A., Leung S.W. (2016). Thirty Years of Saying NO: Sources, Fate, Actions, and Misfortunes of the Endothelium-Derived Vasodilator Mediator. Circ Res..

[79] Alp N.J., Channon K.M. (2004). Regulation of endothelial nitric oxide synthase by tetrahydrobiopterin in vascular disease. Arterioscler Thromb. Vasc. Biol..

[80] Abdelghany T.M., Ismail R.S., Mansoor F.A., Zweier J.R., Lowe F., Zweier J.L. (2018). Cigarette smoke constituents cause endothelial nitric oxide synthase dysfunction and uncoupling due to depletion of tetrahydrobiopterin with degradation of GTP cyclohydrolase. Nitric. Oxide.

[81] Xu J., Wang S., Wu Y., Song P., Zou M.H. (2009). Tyrosine nitration of PA700 activates the 26S proteasome to induce endothelial dysfunction in mice with angiotensin II-induced hypertension. Hypertension.

[82] Xu J., Wu Y., Song P., Zhang M., Wang S., Zou M.H. (2007). Proteasome-dependent degradation of guanosine 5′-triphosphate cyclohydrolase I causes tetrahydrobiopterin deficiency in diabetes mellitus. Circulation.

[83] Lorenz M., Wilck N., Meiners S., Ludwig A., Baumann G., Stangl K., Stangl V. (2009). Proteasome inhibition prevents experimentally-induced endothelial dysfunction. Life Sci..

[84] De Martin R., Hoeth M., Hofer-Warbinek R., Schmid J.A. (2000). The transcription factor NF-kappa B and the regulation of vascular cell function. Arterioscler Thromb. Vasc. Biol..

[85] Takaoka M., Ohkita M., Matsumura Y. (2003). Pathophysiological role of proteasome-dependent proteolytic pathway in endothelin-1-related cardiovascular diseases. Curr. Vasc Pharmacol..

[86] Barroso I., Gurnell M., Crowley V.E., Agostini M., Schwabe J.W., Soos M.A., Maslen G.L., Williams T.D., Lewis H., Schafer A.J. (1999). Dominant negative mutations in human PPARgamma associated with severe insulin resistance, diabetes mellitus and hypertension. Nature.

[87] Savage D.B., Tan G.D., Acerini C.L., Jebb S.A., Agostini M., Gurnell M., Williams R.L., Umpleby A.M., Thomas E.L., Bell J.D. (2003). Human metabolic syndrome resulting from dominant-negative mutations in the nuclear receptor peroxisome proliferator-activated receptor-gamma. Diabetes.

[88] Rahim N.G., Harismendy O., Topol E.J., Frazer K.A. (2008). Genetic determinants of phenotypic diversity in humans. Genome Biol..

[89] Mukohda M., Fang S., Wu J., Agbor L.N., Nair A.R., Ibeawuchi S.C., Hu C., Liu X., Lu K.T., Guo D.F. (2019). RhoBTB1 protects against hypertension and arterial stiffness by restraining phosphodiesterase 5 activity. J. Clin. Invest..

[90] Pelham C.J., Ketsawatsomkron P., Groh S., Grobe J.L., de Lange W.J., Ibeawuchi S.R., Keen H.L., Weatherford E.T., Faraci F.M., Sigmund C.D. (2012). Cullin-3 regulates vascular smooth muscle function and arterial blood pressure via PPARγ and RhoA/Rho-kinase. Cell Metab..

[91] Rivero F., Dislich H., Glockner G., Noegel A.A. (2001). The Dictyostelium discoideum family of Rho-related proteins. Nucleic Acids Res..

[92] Berthold J., Schenkova K., Ramos S., Miura Y., Furukawa M., Aspenstrom P., Rivero F. (2008). Characterization of RhoBTB-depedent Cul3 ubiquitin ligase complexes—evidence for an autoregulatory mechanism. Exp. Cell Res..

[93] Newton-Cheh C., Johnson T., Gateva V., Tobin M.D., Bochud M., Coin L., Najjar S.S., Zhao J.H., Heath S.C., Eyheramendy S. (2009). Genome-wide association study identifies eight loci associated with blood pressure. Nat. Genet..

[94] Evangelou E., Warren H.R., Mosen-Ansorena D., Mifsud B., Pazoki R., Gao H., Ntritsos G., Dimou N., Cabrera C.P., Karaman I. (2018). Genetic analysis of over 1 million people identifies 535 new loci associated with blood pressure traits. Nat. Genet..

[95] Ramos S., Khademi F., Somesh B.P., Rivero F. (2002). Genomic organization and expression profile of the small GTPases of the RhoBTB family in human and mouse. Gene.

[96] Maass P.G., Aydin A., Luft F.C., Schächterle C., Weise A., Stricker S., Lindschau C., Vaegler M., Qadri F., Toka H.R. (2015). PDE3A mutations cause autosomal dominant hypertension with brachydactyly. Nat. Genet..

[97] Chen Y., Yang Z., Meng M., Zhao Y., Dong N., Yan H., Liu L., Ding M., Peng H.B., Shao F. (2009). Cullin mediates degradation of RhoA through evolutionarily conserved BTB adaptors to control actin cytoskeleton structure and cell movement. Mol. Cell.

[98] Ibeawuchi S.R., Agbor L.N., Quelle F.W., Sigmund C.D. (2015). Hypertension-causing Mutations in Cullin3 Protein Impair RhoA Protein Ubiquitination and Augment the Association with Substrate Adaptors. J. Biol. Chem..

[99] Agbor L.N., Ibeawuchi S.C., Hu C., Wu J., Davis D.R., Keen H.L., Quelle F.W., Sigmund C.D. (2016). Cullin-3 mutation causes arterial stiffness and hypertension through a vascular smooth muscle mechanism. JCI Insight..

[100] Abdel Khalek W., Rafael C., Loisel-Ferreira I., Kouranti I., Clauser E., Hadchouel J., Jeunemaitre X. (2019). Severe Arterial Hypertension from Cullin 3 Mutations Is Caused by Both Renal and Vascular Effects. J. Am. Soc. Nephrol..

